# Prognostication in patients with severe traumatic brain injury

**DOI:** 10.7196/AJTCCM.2020.v26i2.076

**Published:** 2020-06-15

**Authors:** Christel Arnold-Day, Patrick L Semple

**Affiliations:** 1 Division of Critical Care, Groote Schuur Hospital, University of Cape Town, South Africa; 2 Division of Neurosurgery, Groote Schuur Hospital, University of Cape Town, South Africa

**Keywords:** Trauma, Brain injury, Post-head injury, Concussion, TBI, Coma

## Editorial

The Hippocratic dictum ‘No head injury is so serious that it should be despaired of, nor so trivial that it can be ignored’ perhaps epitomises the uncertainty that exists around the likely outcome for a patient after sustaining a traumatic brain injury (TBI) most accurately.^[1]^ Despite many advances in modern-day medicine, clinicians’ estimates of prognosis are still often unduly optimistic, unnecessarily cynical, or confusing.^[1]^ Prognosticating on patients who suffer from severe traumatic brain injuries (sTBI) is perhaps only mirrored in its complexity by the nature of the injury they sustained and the pathophysiological consequences thereof.

sTBIs are catastrophic life-altering events that primarily affect young male patients, with mortality rates in the literature ranging from 30 - 50%, while 30% of survivors suffer significant neurological deficit.^[2]^ Population-based studies show that the incidence of TBI is between 180 and 250 per 100 000 population per year in the USA. Incidence is higher in Europe, ranging from 91 per 100 000 in Spain to 546 per 100 000 in Sweden, in South Australia the incidence is 322 per 100 000 and in South Africa (SA) 316 per 100 000.^[3]^ In SA, this equates to ~89 000 new TBI cases being diagnosed and treated annually. These numbers probably underestimate the true incidence of TBI, because they typically only represent the TBI patients admitted to hospital. Many patients with mild TBI (not presenting to a healthcare facility) or with sTBI (associated with death at the scene of the accident or during transport to hospital) may not be accounted for in epidemiological studies. The magnitude of TBIs in Western Cape Province is emphasised by 2013 admissions data from the two tertiary hospitals servicing the province - Groote Schuur Hospital (GSH) and Tygerberg Hospital (TBH); 2 851 patients with TBI were admitted (1 855 at TBH and 996 at GSH). Likewise, an internal audit conducted in 2009 at GSH revealed the high prevalence of TBIs among trauma admissions (n=10 046) where 24% of patients were TBIs, of which 27% (n=654) were classified as moderate to severe and resulted in in-patient admission (King and Webster, unpublished data, 2010).^[4]^

There are also risk factors associated with TBIs. These include age (most common in individuals between the ages of 15 and 40 years with prognosis most guarded in individuals at extremes of age), sex (reported at a ratio of 2 - 4:1 for male:female) and other risk factors such as alcohol and drug abuse.^[3]^ Given the majority of patients are young adults with presumed previous good quality of life, considerable human, social and financial repercussions are experienced by survivors of TBIs with many ‘years of life lost’ (YLL).^[2,5]^ sTBI patients have many potential cognitive, behavioural, psychological and psychiatric consequences that contribute significantly to the national burden of disease, drive high long-term healthcare costs and perpetuate the vicious cycle of violent behaviour.^[5]^

Approximately 50% of TBIs are the result of motor vehicle, bicycle or pedestrian-vehicle accidents, followed by falls (20 - 30% of all TBIs), being more frequent among the elderly and the very young population. Violence-based incidents account for ~20% of TBI, almost equally divided into firearm and non-firearm assaults.^[3]^

Patients who sustained a sTBI need to be fully resuscitated before a prognosis can be attempted. Prognostication is a process that is ever evolving and as patients respond (or not) during resuscitation and initial acute management, their prognosis may continue to change as the attempt to minimise secondary insult to their already vulnerable brain is pursued. It is important to note that a prognosis should not be made on an under-resuscitated patient and life-saving interventions, e.g. a craniotomy, may form part of the resuscitation efforts in the management of these patients. The ubiquitous, although clinically very relevant question from critical care teams regarding a patient’s prognosis is, in most circumstances, rather guarded and the pursuit for a crystal ball answer in TBI management remains ever elusive. There have, however, been many studies done investigating different prognostic schemes, combining clinical signs (pupillary reflexes) with haemodynamic values, intracranial pressure monitor trends and brain oxygen tension parameters, yet most predictive models have thus far been inadequately validated, poorly presented, and based on studies from single centres with small patient numbers that excluded patients from low-income countries (where TBI is most prevalent).^[6]^

Recovery from TBI often necessitates a lengthy and arduous process and may take as long as 12 - 18 months to reach maximum recovery potential. This requires ongoing commitment to treatment and rehabilitation in a system that is often struggling to prioritise and manage limited resources. According to the World Health Organization’s (WHO’s) constitution, health is defined as a state of complete physical, mental and social well-being and not merely the absence of disease or infirmity.^[7]^ Many TBI survivors are left with permanent physical, emotional and cognitive disabilities, thereby falling short on all three WHO constitutional pillars. The neurocognitive and behavioural sequelae of frontal-lobe injuries are often severe, not only for the patient but also for their primary caregivers and may include problems with reasoning, problem solving, information processing, memory, speed of mental processing, judgment, aggression, personality changes, depression, anxiety and reduced social skills.^[3,5]^ The most common of these impairments is short-term memory loss and impulsive behaviour.^[3]^ Dominant temporal lobe injuries can result in communication difficulties due to disorders of speech and language.^[5]^ These deficits have a disabling effect on patients’ ability to fully engage in rehabilitation, to cope with activities of daily living and with constructive engagement within their families and communities. Poor impulse control and weak social skills result in dangerous situations for patients and for those around them. The link between TBI and criminal behaviour is well described in international literature. In a Finnish study, adolescent TBI patients were found to have committed crimes significantly more often than adolescents without a history of a TBI (53.8% v. 14.7%, respectively). Furthermore, the prevalence of both violent crimes (42.9% v. 9.1%), and non-violent crimes (29.4% v. 6.8%) was also higher in the TBI group. The rehabilitation of TBI patients is therefore not only a health issue for patients and their families, but also a critical violence prevention strategy for SA.^[5]^

The impact and complications of TBI are not restricted to neurological consequences. Gastrointestinal complications occur in ~50% of patients; these patients may develop hepatic dysfunction, bowel incontinence and dysphagia, with consequent malnutrition.^[3]^ Musculoskeletal complications may include contractures, osteoporosis and muscle wasting. Other common neurological complications include but are not limited to seizure disorders (post traumatic epilepsy), infections (wound infections, meningitis), nerve damage (cranial nerve injuries) which may result in inability to perceive smell, or control eye movements and post traumatic hydrocephalus. Post concussion syndrome is another complex disorder which may occur in patients after sustaining mild TBI, in which concussion symptoms such as headaches, dizziness, sleeping difficulty, memory problems and irritability manifest and the symptoms may last for weeks, months or even a year after the injury occurred.^[8]^

Management strategies for TBI patients should follow a 3-tiered approach to yield the best results – this should include prevention, evidence-based acute medical management and long-term rehabilitation and support. Prevention strategies typically fall in the realm of healthcare administrators and policymakers, and they include methods such as road safety measures, fall preventions with health and safety acts and legislation on gun safety. Acute medical and surgical management aim to prevent or minimise secondary brain injury and to give the patient the best chance of a good outcome. The third and final tier of the management strategy, arguably the most significant in terms of functional impact to the patient, but also perhaps the most neglected, is the long-term rehabilitation.

As is well illustrated in the article by Maasdorp et al. ^[9]^ in this issue of the AJTCCM, the availability and accessibility of adequate post-hospitalisation rehabilitation for these patients are of paramount importance to ensure better outcomes. In SA, there is limited access to rehabilitation facilities in the public sector. The consequence for TBI patients is that very few receive adequate rehabilitation services; only 2.4% (n=16/654) of the patients in the 2009 Groote Schuur Hospital audit were admitted to a public rehabilitation facility. Over a 5-year period (2008 - 2012), TBI patients made up less than 9% of the Western Cape Rehabilitation Centre (WCRC)’s intake.^[5]^ The WCRC is a 240-bed rehabilitation centre, with daily out-patient services and the only dedicated rehabilitation service available to public patients in the Western Cape. In addition, TBI may include an initial period of post traumatic amnesia and usually involves a slow recovery period, yet candidates for rehabilitation centres are expected to behave appropriately and recover in a 6 - 12-week period, further affecting their ability to gain the maximum service from the necessary rehabilitation support, and thus the majority of patients are instead discharged to unprepared families who typically become their main support structure. This ultimately negatively affects caregiver wellness and family resilience.^[5]^

sTBI patients will typically be cared for by a multi-disciplinary team of healthcare professionals who specialise in the care of TBI patients through rehabilitation. The team typically consists of a physiatrist, physiotherapist, occupational therapist, speech and language therapist, neuropsychologist, rehabilitation nurse and social worker. Their goals are to stabilise the medical and rehabilitation issues related to the TBI and other injuries; prevent secondary complications (pressure sores, pneumonia or contractures); restore lost functional abilities (ability to move, use the bathroom, talk, eat) and to improve and often retrain cognitive processes; to provide adaptive devices or strategies to enhance functional independence; to analyse the patients home circumstance and assess if any home changes need to be made (e.g. access for a wheelchair) for when the patient is ready to go home.

Prediction of outcome involves making probability statements that depend on a rational relationship between outcome and parameters condensed in early data. The advances in prognosis reflect the establishment of methods for categorising outcome and early injury severity. These became widely accepted and led to multinational, multicentre studies that identified characteristics of the patient, the nature of the injury, and the early clinical course with a distinctive and consistent relationship to outcome.^[1]^ sTBI remains a devastating condition that causes major physical, neurocognitive and psychosocial complications and requires a multidisciplinary approach dedicated to long-term rehabilitation and follow-up for treatment to be effective and to ensure the best possible outcomes for the patient.

At least in the context of deciding whether or not to treat individual patients, it is important to continue to acknowledge, as physicist Niels Bohr did, that ‘prediction is very difficult, especially about the future.’^[6]^

## References

[1] Chesnut R, Ghajar J, Maas A, Marion D, Servadei F, Teasdale G (2000). Guidelines for the management and prognosis of severe traumatic brain injury part II: Early indicators
of prognosis in severe traumatic brain injury. J Neurotrauma.

[2] Turgeon AF, Lauzier F, Zarychanski R (2017). Prognostication in critically ill patients
with severe traumatic brain injury: The TBI-prognosis multicentre feasibility study.. BMJ.

[3] National Institute of Occupational Health Traumatic Brain Injury – World Head
Injury Awareness 2013.. http://www.nioh.ac.za/traumatic-brain-injury-head-injuries-world-head-injury-awareness/.

[4] Andrew SF, Rothemeyer S, Balchin R (2017). Improving traumatic brain injury outcomes:
The development of an evaluation and referral tool at Groote Schuur Hospital.. World Neurosurg.

[5] Webster J, Taylor A, Balchin R (2015). Traumatic brain injury, the hidden pandemic:
A focused response to family and patient experiences and needs. S Afr Med J.

[6] Menon D, Harrison D (2008). Prognostic modelling in traumatic brain injury.. BMJ.

[7] World Health Organization WHO Constitution on Health 1946. https://www.who.int/about/who-we-are/constitution.

[8] Ryan LM, Warden DL (2003). Post concussion syndrome. Int Rev Psychiatr.

[9] Maasdorp SD, Swanepoel C, Gunter L (2020). Outcomes of severe traumatic brain injury at the time of discharge from tertiary academic hospitals in Bloemfontein. Afr J Thoracic Crit Care Med.

